# Short-Term Effects of Nitrogen Dioxide on Mortality and Susceptibility Factors in 10 Italian Cities: The EpiAir Study

**DOI:** 10.1289/ehp.1002904

**Published:** 2011-05-17

**Authors:** Monica Chiusolo, Ennio Cadum, Massimo Stafoggia, Claudia Galassi, Giovanna Berti, Annunziata Faustini, Luigi Bisanti, Maria Angela Vigotti, Maria Patrizia Dessì, Achille Cernigliaro, Sandra Mallone, Barbara Pacelli, Sante Minerba, Lorenzo Simonato, Francesco Forastiere, on behalf of the EpiAir Collaborative Group

**Affiliations:** 1 Environmental Epidemiological Unit, Regional Environmental Protection Agency, Piedmont, Turin, Italy; 2 Department of Epidemiology, Rome E Local Health Authority, Rome, Italy; 3 Cancer Epidemiology Unit, Center for Cancer Prevention, Piedmont, Turin, Italy; 4 Epidemiology Unit, Local Health Authority, Milan, Italy; 5 Department of Biology, University of Pisa, Pisa, Italy; 6 Department of Prevention, Local Health Authority, Cagliari, Italy; 7 Epidemiological Observatory, Regional Health Authority, Palermo, Italy; 8 Institute for Cancer Study and Prevention, Florence, Italy; 9 Epidemiology Observatory, Department of Public Health, Local Health Authority, Bologna, Italy; 10 Statistics and Epidemiology Unit, Local Health Authority, Taranto, Italy; 11 Department of Environmental Medicine and Public Health, School of Medicine, University of Padova, Padova, Italy

**Keywords:** case-crossover analysis, environmental epidemiology, mortality, nitrogen dioxide, outdoor air pollution, short-term exposure, susceptibility

## Abstract

Background: Several studies have shown an association between nitrogen dioxide (NO_2_) and mortality. In Italy, the EpiAir multicentric study, “Air Pollution and Health: Epidemiological Surveillance and Primary Prevention,” investigated short-term health effects of air pollution, including NO_2_.

Objectives: To study the individual susceptibility, we evaluated the association between NO_2_ and cause-specific mortality, investigating individual sociodemographic features and chronic/acute medical conditions as potential effect modifiers.

Methods: We considered 276,205 natural deaths of persons > 35 years of age, resident in 10 Italian cities, and deceased between 2001 and 2005. We chose a time-stratified case-crossover analysis to evaluate the short-term effects of NO_2_ on natural, cardiac, cerebrovascular, and respiratory mortality. For each subject, we collected information on sociodemographic features and hospital admissions in the previous 2 years. Fixed monitors provided daily concentrations of NO_2_, particulate matter ≤ 10 μm in aerodynamic diameter (PM_10_) and ozone (O_3_).

Results: We found statistically significant associations with a 10-μg/m^3^ increase of NO_2_ for natural mortality [2.09% for lag 0–5; 95% confidence interval (CI), 0.96–3.24], for cardiac mortality (2.63% for lag 0–5; 95% CI, 1.53–3.75), and for respiratory mortality (3.48% for lag 1–5; 95% CI, 0.75–6.29). These associations were independent from those of PM_10_ and O_3_. Stronger associations were estimated for subjects with at least one hospital admission in the 2 previous years and for subjects with three or more specific chronic conditions. Some cardiovascular conditions (i.e., ischemic heart disease, pulmonary circulation impairment, heart conduction disorders, heart failure) and diabetes appeared to confer a strong susceptibility to air pollution.

Conclusions: Our results suggest significant and likely independent effects of NO_2_ on natural, cardiac, and respiratory mortality, particularly among subjects with specific cardiovascular preexisting chronic conditions and diabetes.

Nitrogen dioxide (NO_2_) is a strong respiratory irritant gas originating from high-temperature combustion. Main outdoor sources of NO_2_ include motor vehicles (particularly those equipped with diesel engines) and fossil-fuel power plants, whereas the most important indoor sources are gas heaters, stoves, and environmental tobacco smoke [Kraft et al. 2005; U.S. Environmental Protection Agency (EPA) 2008].

Large meta-analyses of studies on the short-term health effects of NO_2_ have been carried out in Europe (Samoli et al. 2006; Touloumi et al. 1997; Zmirou et al. 1998), the United States (Stieb et al. 2002, 2003), and Canada (Shin et al. 2008). The results indicate a positive association between daily increases of NO_2_ and natural, cardiovascular, and respiratory mortality. The findings are consistent with an independent effect of NO_2_, although the possibility remains that NO_2_ acts as a surrogate for other unmeasured pollutants (Samoli et al. 2006). Several epidemiological studies have indicated that NO_2_ may be a more relevant health-based exposure indicator than particulate matter (PM) (Kan and Chen 2003; Sarnat et al. 2001; Schwartz et al. 1994). Based on these observations, the U.S. EPA has recently proposed to strengthen the NO_2_ air quality standard that protects public health (U.S. EPA 2008).

Despite the large body of evidence linking NO_2_ with daily mortality, few studies have addressed the issue of susceptibility to NO_2_ by performing analyses by age, sex, and other factors, including socioeconomic status (SES) (Laurent et al. 2007) and chronic morbidity. On the other hand, the evaluation of the role of susceptibility factors in modifying the effect of air pollutants is of increasing interest in order to better understand the mechanisms of NO_2_ health effects and to provide public health warnings to specific population subgroups. Along these lines, we have already explored the role of individual characteristics (age, sex, socioeconomic factors, and clinical characteristics) as effect modifiers of the association of PM ≤ 10 μm in aerodynamic diameter (PM_10_) and ozone (O_3_) with natural mortality (Forastiere et al. 2008; Stafoggia et al. 2010). The present study is part of EpiAir, the Italian surveillance project on the health effects of air pollution whose main objective is the continuous update of the effect estimates for PM_10_, NO_2_, and O_3_ (Berti et al. 2009b).

The specific objectives of the present article were to investigate the NO_2_–mortality relationship for specific causes of death while exploring the latency of the effects and the potential confounding role by other pollutants and to evaluate sociodemographic features and chronic or acute medical conditions as potential effect modifiers. Preliminary results of this study were presented at the 2009 Conference of the International Society for Environmental Epidemiology (Chiusolo et al. 2009).

## Materials and Methods

*Health data.* We collected mortality data for 10 Italian cities (Bologna, Cagliari, Florence, Mestre–Venice, Milan, Palermo, Pisa, Rome, Taranto, and Turin); this data accounted for about 12% of the total Italian population (Table 1). We selected 276,205 subjects ≥ 35 years old, resident within the city at the time of death, who died between 2001 and 2005 of natural causes [*International Classification of Diseases*, version 9 (ICD-9), codes 1–799 (World Health Organization 1979)]. The underlying cause of death was classified as cardiac (ICD-9 codes 390–429), cerebrovascular (ICD-9 codes 430–438), and respiratory (ICD-9 codes 460–519). The resident population data (year 2001) were recovered from the census office registry.

**Table 1:** Study period, total population, and number and percentage of subjects who resided and died in 10 Italian cities: EpiAir Study, Italy, 2001–2005.

Study period	Total population (*n*)*a*	Cardiac mortality		Cerebrovascular mortality		Respiratory mortality		All natural mortality	
		City	*n* (%)		*n* (%)		*n* (%)		*n* (%)	
Bologna		2001–2005		371,217		5,581	(27.5)		1,888	(9.3)		1,719	(8.5)		20,314	(100.0)
Cagliari		2002–2005		164,249		1,228	(24.1)		585	(11.5)		463	(9.1)		5,094	(100.0)
Florence		2001–2005		356,118		4,383	(25.9)		1,744	(10.3)		1,450	(8.6)		16,940	(100.0)
Mestre–Venice		2001–2005		195,790		2,698	(29.7)		910	(10.0)		421	(4.6)		9,076	(100.0)
Milan		2001–2005		1,256,211		13,021	(25.2)		5,383	(10.4)		4,391	(8.5)		51,736	(100.0)
Palermo		2002–2005		686,722		5,277	(24.8)		2,327	(10.9)		1,404	(6.6)		21,320	(100.0)
Pisa		2001–2005		89,694		1,225	(27.5)		585	(13.2)		361	(8.1)		4,447	(100.0)
Rome		2001–2005		2,546,804		31,895	(30.8)		9,684	(9.3)		6,077	(5.9)		103,677	(100.0)
Taranto		2001–2005		202,033		1,759	(25.5)		642	(9.3)		562	(8.2)		6,885	(100.0)
Turin		2001–2005		865,263		9,376	(25.5)		4,732	(12.9)		2,781	(7.6)		36,716	(100.0)
Total		2001–2005		6,734,101		76,443	(27.7)		28,480	(10.3)		19,629	(7.1)		276,205	(100.0)
**a**Population at 2001 census.																

For all centers except Cagliari (where hospital discharge data were not available at the time of the study), we collected data at the individual level on the following susceptibility factors: age, sex, median income of the census block of residence (these data were available only for Milan, Turin, Bologna, and Rome and accounted for 75% of the study population), and median socioeconomic position of the census block of residence (these data were available only for Mestre–Venice, Pisa, Rome, Taranto, and Turin and accounted for 44% of the study population). A record linkage with the regional archives of hospital admission databases allowed us to gather data on the place of death [classified as out-of-hospital, recently discharged (within 4 weeks) from a hospital, in-hospital, nursing home] and on discharge diagnoses in the previous 2 years.

Health conditions in the 24 months before death were classified as “chronic” or “acute” according to several criteria. Chronic diseases were those with a course consistent with clinical criteria of chronicity that were diagnosed at least 1 month before death and did not present a recent exacerbation; both primary and secondary discharge diagnoses were considered for hospitalizations that occurred between 29 days and 2 years before death. Acute conditions included not only clinical manifestations with sudden onset, short course, and high likelihood to be cured but also exacerbations of chronic diseases, provided that both these clinical forms caused a hospitalization within 1 month before death; only the primary discharge diagnoses were considered for hospitalizations that occurred in the 4 weeks before death. We based our list of diagnoses on Elixhauser’s list of comorbidities (Elixhauser et al. 1998); this approach is consistent with previous work (Forastiere et al. 2008). We selected as “chronic conditions” diabetes, coagulation disorders, hypertension, myocardial infarction, cardiac ischemic diseases, diseases of pulmonary circulation, heart conduction disorders, dysrhythmias, heart failure, cerebrovascular diseases, and chronic pulmonary diseases; “acute conditions” included diseases of pulmonary circulation, dysrhythmias, heart failure, and renal failure.

*Environmental data.* Air pollution data were provided through city-specific air monitoring networks managed by regional environmental agencies or local authorities. We obtained data on nitrogen dioxide (NO_2_; daily average, micrograms per cubic meter), ozone (O_3_; daily maximum 8-hr running mean, micrograms per cubic meter), and PM_10_ (daily average, micrograms per cubic meter) (Table 2). Air pollution data were collected according to methods already employed in several European studies. We estimated daily levels of air pollutants for each city by averaging monitor-specific daily measurements available from different monitoring stations. A previously defined algorithm was implemented to impute missing values for pollutant concentrations in each center (Berti et al. 2009a; Biggeri et al. 2004).

**Table 2:** Descriptive characteristics of air pollutants by city: EpiAir Study, Italy, 2001–2005.

Daily mean NO_2_ (μg/m^3^)							Daily mean PM_10_ (μg/m^3^)							Daily maximum O_3_ from 8-hr running means (μg/m^3^)*a*						
City	No. monitors	Mean ± SD	50th percentile	90th percentile	No. monitors	Mean ± SD	50th percentile	90th percentile	No. monitors	Mean ± SD	50th percentile	90th percentile
Bologna		3		52 ± 18		50		75		1		43 ± 25*b*		36*b*		76*b*		2		91 ± 31		89		131
Cagliari		2		34 ± 16		33		54		3		32 ± 12		30		48		3		81 ± 19*c*		79*c*		108*c*
Florence		3		46 ± 19		44		68		4		38 ± 18		35		61		3		96 ± 24		96		125
Mestre–Venice		3		38 ± 14		36		58		2		48 ± 33*b*		39*b*		88*b*		3		91 ± 30		88		131
Milan		3		59 ± 23		57		88		5		52 ± 32		43		95		2		91 ± 34		89		138
Palermo		3		52 ± 16		51		74		3		35 ± 19		32		52		2		87 ± 18		86		111
Pisa		3		30 ± 11		29		45		3		34 ± 15		31		53		1		99 ± 21		99		127
Rome		3		62 ± 16		62		82		3		39 ± 16		37		59		2		105 ± 25		103		140
Taranto		4		26 ± 11		24		41		2		50 ± 21*d*		48*d*		81*d*		3		78 ± 21		78		104
Turin		3		66 ± 20		64		92		2		55 ± 34*b*		44*b*		102*b*		1		115 ± 39		113		170
**a**Data for O_3_ from April–September. **b**Data availability 2002–2005. **c**Data availability 2003–2005. **d**Data availability 2001–2004.																								

We collected data on meteorological variables (air temperature, dew point temperature, and barometric pressure) from the Italian Air Force Meteorological Service. Apparent temperature was estimated taking into account air temperature and humidity (Kalkstein and Valimont 1986).

*Statistical analysis.* We investigated the association between NO_2_ and mortality using a case-crossover design (Maclure 1991). To control for time trends, we selected control days using a time-stratified approach (Levy et al. 2001); the study period was divided into monthly strata, and the control days for each case were chosen on the same day of the week in the stratum. We applied a conditional logistic regression to data from each city. We considered as confounding factors: population decrease, holidays, influenza epidemics, barometric pressure, and apparent temperature. Day of the week and long-term and seasonal trends were adjusted for by design. For population decreases during summer vacation periods (typical of Italian cities, when urban populations are substantially reduced), we defined a three-level variable: 2 for the 2-week period around mid-August, 1 for the time period from 16 July to 31 August (with the exception of the mentioned 2-week period), and 0 (the reference category) otherwise. For holidays we defined a two-level variable: 1 for national or city-specific holidays, and 0 otherwise. For influenza epidemics we defined a two-level variable: 1 for the 3-week winter period of influenza epidemic peak (as defined for each year by the National Institute of Health), and 0 for the remaining days of the year. For barometric pressure we used a penalized spline of the original variable at lag 0. The effect of temperature on mortality was controlled for by modeling high temperatures and low temperatures separately. More specifically, high temperatures were adjusted for by calculating the average of current- and previous-day apparent temperature (lag 0–1) and by fitting a penalized spline of the lagged variable only for days with lag 0–1 apparent temperature above the city-specific median value, calculated on the year-round time series of apparent temperature. Similarly, low-winter temperatures were adjusted for by calculating the mean air temperature of the previous 6 days (lag 1–6) and by fitting a penalized spline of the lagged variable only for days with lag 1–6 air temperature below the city-specific median value, as determined from the year-round series.

We began by modeling the effect of NO_2_ on cause-specific mortality for each city using unconstrained distributed lag models, cubic polynomial distributed lag models, and single-lag models to identify the best lag structure. Unconstrained distributed lag models were implemented for different lag structures *a priori*, defined as immediate (up to lag 1), delayed (lag 2–5), and prolonged (lag 0–5) effects, with the aim of obtaining an unbiased estimate of the cumulative effect of NO_2_ on mortality at the different lags. Cubic polynomial distributed lag models were fitted within each city in order to visually display the latency of the NO_2_ effect, being aware that single days of the polynomial curve were not directly interpretable in terms of effect size. Finally, we fitted single-lag models in order to estimate the effect of NO_2_ on mortality for each specific exposure day separately, up to 5 days before death. All the city-specific results (from distributed-lag and single-lag models) were finally pooled to estimate the lagged effect of NO_2_ across all of the cities. Pooled estimates were obtained from city-specific results by applying a random-effects meta-analysis (maximum likelihood method) (Normand 1999; van Houwelingen et al. 2002).

We implemented bipollutant models in order to estimate the association of NO_2_ with cause-specific mortality while adjusting for PM_10_ or, in turn, O_3_ (the latter during April–September only). The lag exposures were chosen within the unconstrained distributed-lag modeling framework, selecting the lags showing the strongest association. A pooled estimate was obtained from city-specific results via random-effects meta-analysis (Normand 1999; van Houwelingen et al. 2002).

We evaluated the role of the potential effect modifiers (sex, age, SES at the census block level, chronic and acute conditions as previously defined) via conditional logistic regression models stratified by the levels of each presumed effect modifier: we compared the effect estimate for NO_2_ in each category of the potential effect modifier with the effect estimate in the reference category for the potential modifier, from the stratified models. Because all effect modifiers were likely to be associated with age (e.g., chronic conditions), all stratum-specific estimates were standardized by age, using the relative frequencies of the overall age distribution as weights. We formally evaluated statistical significance (at α = 0.05 level) of the effect modification and computed *p*-values for relative effect modification (*p*-REM). In particular, the relative effect modification (REM) was evaluated by analyzing the difference between the coefficient of the NO_2_–mortality association within a specific stratum of the effect modifier and the coefficient within the reference stratum of the same variable. The corresponding *p*-value (*p*-REM) is derived by assuming that the difference between the two coefficients follows a normal distribution with zero mean and variance equal to the sum of the two stratum-specific variances. We assumed that effect modification was “likely” when *p*-REM ≤ 0.05, regardless of the magnitude of the stratum-specific association estimate. The effect modification was “suggested” when 0.05 < *p*-REM < 0.20 and either *a*) the risk estimated in a specific stratum was twice the risk estimated in the reference stratum or *b*) the excess risk estimated for the stratum was statistically significant. In addition, in case of a possible effect modifier with more than two ordinary modalities (e.g., number of chronic condition), we considered evidence of effect modification to be “suggestive” when a dose–response trend was observed.

Analysis of effect modification by individual characteristics was performed for each city, and pooled effects were estimated via random-effect meta-analysis (Normand 1999). For each pooled effect estimate, we computed the *Q*-statistic and the *p*-value of heterogeneity (HET) to test for heterogeneity among city-specific estimates (against the null hypothesis that the city-specific estimates were homogeneous).

We express all effect estimates as the percent increase in mortality, with corresponding 95% confidence intervals (CIs), associated with a 10-μg/m^3^ increase in NO_2_.

We performed the statistical analyses using SAS (version 8.0; SAS Institute Inc., Cary, NC, USA) and R software (version 2.6.1; R Project for Statistical Computing, Vienna, Austria).

## Results

Table 1 summarizes population and mortality data for each city included in the analysis. We considered a total of 276,205 natural (nonaccidental) deaths among those > 35 years of age. Cardiac, cerebrovascular, and respiratory mortality accounted for about 28%, 10%, and 7% of natural deaths, respectively.

Table 2 summarizes the descriptive statistics for air pollution indicators, expressed as daily means over the time period considered, for each city. For NO_2_, values consistently exceeded 40 μg/m^3^ in six cities.

**Figure 1:**
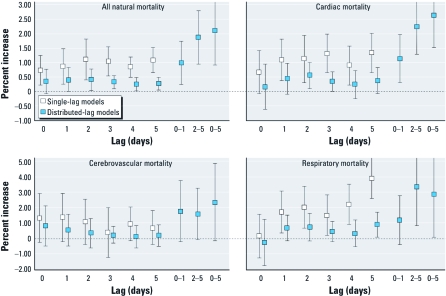
NO_2_ and mortality, by cause of death and lag (single-lag and constrained and unconstrained distributed-lag models). Values shown are percent increases of risk (95% CI) for 10-μg/m^3^ increases in NO_2_ (pooled results from 10 cities), EpiAir Study, Italy, 2001–2005.

Figure 1 shows pooled effect estimates (10 cities) for the association between NO_2_ and mortality by cause of death and lag (single-lag models and constrained and unconstrained distributed-lag models). The lag structure suggests a prolonged effect of the NO_2_ on all outcomes considered up to lag 5, whereas a delayed association was more evident for respiratory mortality, from lag 1 to 5. Based on these results, we selected lag 0–5 as the lag with the maximum estimated effect for natural, cardiac, and cerebrovascular mortality, and lag 1–5 as the lag with the maximum estimated effect for respiratory mortality.

Pooled results for all 10 cities indicated that a 10-μg/m^3^ increase in NO_2_ was significantly associated (at α = 0.05 level) with all natural mortality, cardiac mortality, and respiratory mortality, with the strongest estimated effects for respiratory mortality (Table 3). The estimated effects of NO_2_ were not confounded by PM_10_ in bipollutant models. Associations were enhanced during the warm season (April–September) when the NO_2_–cerebrovascular disease association also became statistically significant, but these associations were not confounded by O_3_. Effect estimates for natural and respiratory mortality were heterogeneous across cities, with Rome being an outlier for natural mortality (4.41%; 95% CI, 3.38–5.45) and Turin for respiratory mortality (–1.83%; 95% CI, –3.35 to –0.28). No significant heterogeneity was present for cardiac and cerebrovascular outcomes.

**Table 3:** Percent increase in risk of death (95% CI) for a 10-μg/m^3^ increase in NO_2_: single-pollutant models and models adjusted for PM_10_ and O_3_ (pooled results, 10 cities), EpiAir Study, Italy, 2001–2005.

Model	Percent increase in risk	95% CI	HET*a*
All natural mortality						
NO_2_ (lag 0–5), single-pollutant model		2.09		0.96 to 3.24		0.001
Model with PM_10_ (lag 0–5)		1.95		0.50 to 3.43		0.003
April–September						
NO_2_ (lag 0–5), single-pollutant model		4.46		3.14 to 5.80		0.109
Model with O_3_ (lag 0–5)		4.55		3.32 to 5.79		0.175
Cardiac mortality						
NO_2_ (lag 0–5), single-pollutant model		2.63		1.53 to 3.75		0.658
Model with PM_10_ (lag 0–5)		2.58		1.05 to 4.13		0.265
April–September						
NO_2_ (lag 0–5), single-pollutant model		4.77		2.92 to 6.65		0.871
Model with O_3_ (lag 0–5)		4.69		2.74 to 6.67		0.925
Cerebrovascular mortality						
NO_2_ (lag 0–5), single-pollutant model		2.35		–0.13 to 4.89		0.266
Model with PM_10_ (lag 0–5)		2.55		–0.71 to 5.92		0.247
April–September						
NO_2_ (lag 0–5), single-pollutant model		7.87		4.78 to 11.05		0.628
Model with O_3_ (lag 0–5)		7.26		3.51 to 11.14		0.420
Respiratory mortality						
NO_2_ (lag 1–5), single-pollutant model		3.48		0.75 to 6.29		0.000
Model with PM_10_ (lag 0–5)		3.39		0.77 to 6.08		0.512
April–September						
NO_2_ (lag 1–5), single-pollutant model		9.63		4.08 to 15.47		0.016
Model with O_3_ (lag 0–5)		10.07		3.69 to 16.83		0.004
**a***p*-Value from the heterogeneity test (the null hypothesis being homogeneity of the city-specific results).						

Pooled associations between NO_2_ and natural mortality (lag 0–5) are reported for nine cities (excluding Cagliari), overall and according to strata of selected susceptibility factors (Table 4). We observed an overall increase of 2.03% (95% CI, 0.87–3.21) in natural mortality associated with a 10-μg/m^3^ increase of NO_2_. The association was stronger for subjects > 84 years of age (3.41%; 95% CI, 2.10–4.74) than for younger subjects, but age was not a significant effect modifier (*p*-REM = 0.270), and associations did not follow a monotonic trend with age. Neither income nor socioeconomic position (both measured as the median of the census block of residence) significantly modified the association between NO_2_ and mortality, but we observed significant heterogeneity in the stratum-specific effect estimates among the cities.

**Table 4:** NO_2_ and all natural mortality among subjects ≥ 35 years of age who resided and died in nine Italian cities, pooled results by sociodemographic characteristic and chronic condition, EpiAir Study, Italy, 2001–2005.

Variable	*n* (%)		Percent increase in risk	95% CI	*p*-REM*a*	HET*b*
All natural deaths (≥ 35 years of age, lag 0–5)		271,111	(100.0)		2.03	0.87 to 3.21		—		0.001
Age (years)										
35–64		35,803	(13.2)		2.17	0.42 to 3.95		—		0.022
65–74		52,689	(19.4)		0.42	–2.14 to 3.04		0.275		0.000
75–84		92,539	(34.1)		1.91	0.71 to 3.11		0.809		0.340
≥ 85		90,070	(33.2)		3.41	2.10 to 4.74		0.270		0.096
Sex*c*										
Men		130,428	(48.1)		2.35	1.36 to 3.35		—		0.171
Women		140,674	(51.9)		1.71	–0.16 to 3.61		0.556		0.000
SES (average of the census tract)*c*^,d^										
Low (< 20th percentile)		33,565	(12.4)		3.09	0.90 to 5.32		—		0.326
Middle (20th to 80th percentile		93,040	(34.3)		1.70	–0.12 to 3.55		0.343		0.029
High (> 80th percentile)		32,277	(11.9)		2.50	–0.46 to 5.55		0.758		0.001
Income (average of the census tract)*c*^,e^										
Low (< 20th percentile)		47,721	(17.6)		2.95	0.32 to 5.65		—		0.008
Middle (20th to 80th percentile)		122,394	(45.1)		3.01	1.75 to 4.28		0.969		0.023
High (> 80th percentile)		39,681	(14.6)		1.33	–0.74 to 3.43		0.347		0.160
Location of death*c*										
Outside the hospital, not hospitalized during the last 4 weeks		103,538	(38.2)		1.63	0.24 to 3.04		—		0.012
Outside the hospital, hospitalized during the last 4 weeks		25,778	(9.5)		2.97	0.98 to 4.99		0.284		0.349
In hospital*f*		132,181	(48.8)		2.48	1.12 to 3.86		0.396		0.107
In a nursing home*g*		9,611	(3.5)		0.89	–0.246 to 4.34		0.691		0.498
Season of death*c*										
October–March		144,464	(53.3)		1.18	0.20 to 2.16		—		0.006
April–September		126,647	(46.7)		4.64	3.33 to 5.97		0.000		0.128
Hospital admission between 0 and 28 days before death*c*										
No		149,953	(55.3)		1.82	0.56 to 3.09		—		0.021
Yes		121,158	(44.7)		2.56	1.16 to 3.97		0.442		0.055
Hospital admission between 29 days and 2 years before death*c*										
No		95,096	(35.1)		0.73	–0.69 to 2.18		—		0.024
Yes		176,015	(64.9)		2.86	1.39 to 4.35		0.043		0.009
No. of specific chronic conditions*c*^,h^										
0		152,100	(56.1)		1.54	0.27 to 2.82		—		0.001
1		41,547	(15.3)		2.39	0.03 to 4.80		0.536		0.046
2		32,390	(11.9)		2.99	0.49 to 5.55		0.313		0.188
≥ 3		45,074	(16.6)		3.62	2.04 to 5.22		0.045		0.720
Specific chronic conditions*c*^,h ^(ICD-9 code)										
Diabetes (250)		30,620	(11.3)		3.61	1.73 to 5.53		0.108		0.954
Coagulation disorders (286, 287)		3,285	(1.2)		3.26	–3.66 to 10.68		0.699		0.438
Hypertension (401–405)		53,441	(19.7)		2.24	0.85 to 3.65		0.752		0.283
Myocardial infarction (410, 412)		12,828	(4.7)		3.30	0.41 to 6.27		0.367		0.489
Cardiac ischemic diseases (410–414)		37,225	(13.7)		3.22	1.52 to 4.96		0.185		0.508
Diseases of pulmonary circulation (415–417)		5,269	(1.9)		8.03	3.17 to 13.12		0.014		0.649
Heart conduction disorders (426)		6,213	(2.3)		5.91	1.78 to 10.20		0.064		0.540
Dysrhythmias (427)		34,529	(12.7)		3.43	0.88 to 6.04		0.243		0.115
Heart failures (428)		28,174	(10.4)		3.40	1.43 to 5.40		0.166		0.283
Cerebrovascular diseases (430–438)		36,937	(13.6)		2.73	0.16 to 5.38		0.548		0.321
Chronic pulmonary diseases (490–505)		32,859	(12.1)		3.05	1.21 to 4.92		0.260		0.331
—, Reference category. Data include number and percentage of subjects ≥ 35 years of age and percent increases of risk of death for natural causes (95% CI) for a 10-μg/m^3^ increase in NO_2_; pooled results by age, sex, indicators of SES, location of death, season, previous hospitalizations, and specific chronic conditions (nine cities, except Cagliari). **a***p*-Value of REM, derived from the difference between the coefficient of the stratum and the coefficient of the reference category (for each chronic condition, the reference category is the group of subjects without the disease). **b***p*-Value of heterogeneity test (null hypothesis representing perfect homogeneity of city-specific results). **c**Results standardized by age, with weights equal to relative frequencies of subjects in the age groups 35–64, 65–74, 75–84, and ≥ 85 years, from the nine cities analyzed. **d**Data available only for Mestre–Venice, Pisa, Rome, Taranto, and Turin. **e**Data available only for Bologna, Milan, Rome, and Turin. **f**Also includes subjects who died outside the hospital but were discharged the same day of death or the previous day. **g**Data available only for Florence, Milan, and Turin. **h**Chronic conditions are based on primary or secondary contributing diagnoses of any hospital admissions occurred between 29 days and 2 years before death.										

The season of death significantly modified the association between NO_2_ and all natural mortality, with a stronger association (4.64%; 95% CI, 3.33–5.97) during the warm season than during the rest of the year (1.18%; 95% CI, 0.20–2.16; *p*-REM = 0.000). The association between NO_2_ and all natural mortality also was significantly stronger among subjects with at least one hospital admission between 2 years and 29 days before death (2.86%; 95% CI, 1.39–4.35) than among other subjects (0.73%; 95% CI, –0.69 to 2.18; *p*-REM = 0.043). Similarly, the association was significantly stronger for subjects with three or more chronic conditions (3.62%; 95% CI, 2.04–5.22) than for those without chronic conditions (1.54%; 95% CI, 0.27–2.82; *p*-REM = 0.045), with a monotonic increase in the excess risk in relation to the number of chronic conditions. Associations between NO_2_ and all natural mortality also were stronger among subjects hospitalized between 2 years and 29 days before death for the specific chronic conditions examined. Estimated risks were particularly high for subjects with disorders of pulmonary circulation (8.03%; 95% CI, 3.17–13.2; *p*-REM = 0.014), and we found some evidence of effect modification by heart conduction disorders (5.91%; 95% CI, 1.78–10.2; *p*-REM = 0.064), diabetes (3.61%; 95% CI, 1.73–5.53; *p*-REM = 0.108), heart failure (3.40%; 95% CI, 1.43–5.40; *p*-REM = 0.166), and cardiac ischemic diseases (3.22%; 95% CI, 1.52–4.96; *p*-REM = 0.185).

We did not find evidence of effect modification by sex, place of death, hospital admissions between 0 and 28 days before death (Table 4), or hospitalization for diseases of the pulmonary circulation, dysrhythmias, heart failure, or renal failure during the 28 days before death (data not shown).

## Discussion

In this study we found statistically significant increases in mortality due to natural, cardiac, and respiratory causes associated with a 10-μg/m^3^ increase in NO_2_ regardless of season, and a significant association between NO_2_ and cerebrovascular mortality during the warm season. Overall, associations were strongest for exposures lagged 0–5 days and were stronger in the warm than in the cold season. Associations with NO_2_ appeared to be independent of PM_10_ and independent of O_3_ exposure during the warm season. Associations with total mortality were stronger for subjects with a hospital admission in the 2 preceding years. Interestingly, previous cardiovascular morbidity (changes in pulmonary circulation, heart conduction disorders, heart failure, and ischemic heart diseases) and diabetes appeared to confer a strong susceptibility.

Excess risks estimated in the present study for a 10-μg/m^3^ increase in NO_2_ (natural causes, lag 0–5: 2.09%; 95% CI, 0.96–3.24; cardiac causes, lag 0–5: 2.63%; 95% CI, 1.53–3.65; respiratory causes, lag 1–5: 3.48%; 95% CI, 0.75–6.29) are higher than those published in previous meta-analyses, although comparisons are limited because of the use of different statistical methods, lags, populations, and metrics for NO_2_ exposure.

Samoli et al. (2006) used the most extensive European database available [Air Pollution on Health: A European Approach (APHEA-2)] to investigate the effects of NO_2_ on mortality. They estimated 0.30%, 0.40%, and 0.38% excess risks for natural, cardiovascular, and respiratory causes, respectively (for a 10-μg/m^3^ increase in NO_2_). The study was related to the calendar period 1990–1997, and the study population was not restricted to a specific age group. The meta-analysis of the Italian studies on short-term effects of air pollution (MISA2) reported at lag 0–1, 0.59%, 0.40%, and 0.38% excess risks for natural, cardiovascular and respiratory causes, respectively (for a 10-μg/m^3^ increase in NO_2_) for deaths during 1996–2002 (Biggeri et al. 2004). Our estimates for deaths in many of the same cities during 2001–2005 are almost double (0.99%, 1.13%, and 1.19% for lag 0–1, respectively) the MISA2 estimates, despite the fact that in recent years the concentrations of NO_2_ are slightly reduced in the cities involved in these Italian studies (Berti et al. 2009a). An interesting temporal and multicity analysis conducted in Canada has suggested that, despite decreasing ambient concentrations over time, mortality risks associated with NO_2_ appear to be increasing (Shin et al. 2008). One possible explanation for our findings and the Canadian results is that NO_2_ itself may not be causally linked to mortality and that the truly toxic components may not be changing over time, at least not at the same rate as NO_2_.

In our study the estimated effects of NO_2_ appeared to extend over the 5 days before death; the shape of the association between lagged NO_2_ and cardiac mortality was comparable to that for natural mortality, with strongest associations with NO_2_ 2 days before death. NO_2_ on the day of death was not associated with respiratory mortality, whereas NO_2_ was 1–5 days before death. This is in agreement with the APHEA-2 results (Samoli et al. 2006).

Analyses restricted to April–September showed stronger associations, with excess risk estimates of 4.49%, 4.77%, and 10.07% for natural, cardiac, and respiratory mortality, respectively; moreover, the risk for cerebrovascular diseases became significant (7.87%) in the warm season. The season of death was, in fact, the strongest of the effect modifiers examined for natural mortality (4.64% during the warm period vs. 1.18% for October–March; *p*-value of REM < 0.001), and these associations persisted after adjustment for O_3_. These results are in agreement with previously published European meta-analyses (Biggeri et al. 2004; Samoli et al. 2006). There can be several possible explanations for this finding, not mutually exclusive. A likely explanation is that during the warm season the measured concentrations of the air pollutants are more representative of the true exposure of the subjects: during the summer people spend more time outside than during the cold season and are more likely to keep windows open, thus allowing ambient air pollutants to enter buildings. Furthermore, an increased individual susceptibility to the effects of air pollutants in summer may be present (Pekkanen et al. 2000).

However, other interpretations cannot be ruled out. First of all, a true synergic effect between NO_2_ and high summer temperature could be present, because that biochemical reaction is faster at higher temperature (Atkins and de Paula 2006). In addition, the summer effect of NO_2_ could be enhanced by the lower average mortality during summer months, given that in Italian cities the dose–response curve is steeper at lower mortality and concentration values (Biggeri et al. 2009).

Our results suggest an effect of NO_2_ on mortality independent from PM_10_, but the role of NO_2_ as a surrogate of unmeasured pollutants cannot be ruled out. In the presence of high levels of traffic, PM consists of a mixture of carbon particles (including PM in the ultrafine range), which are mainly produced by diesel engines (diesel soot). In fact, diesel engines are the main sources of both NO_2_ and ultrafine PM. These ultrafine particles are sulfated and nitrogenized, which may explain the high correlation observed among PM, NO_2_, and sulfur dioxide. From this perspective, NO_2_ should be considered mainly as a surrogate of ultrafine PM (Sarnat et al. 2001). In our EpiAir study, NO_2_ is a strong confounder of the relationship between PM_10_ and mortality: in the single-pollutant model, we observed a significant increase of 0.80% (lag 0–2) for the association of PM_10_ with natural mortality that decreased to 0.18% (not significant) in the bipollutant model with NO_2_. This is consistent with the results of a previous Italian meta-analysis (Biggeri et al. 2004).

Specific groups within the general population are at increased risk of adverse effects from NO_2_ exposure. Factors that may influence susceptibility to the effects of air pollution include age (e.g., elderly) (Liu et al. 2007; Yang et al. 2006), sex, race/ethnicity, genetic factors, and preexisting diseases or conditions (e.g., obesity, diabetes, respiratory disease, asthma, chronic obstructive pulmonary disease, cardiovascular disease, dysrhythmias, airway hyperresponsiveness, respiratory infection) (Dales et al. 2006; Felber Dietrich et al. 2008; Hoek et al. 2000). In addition, exposure to air pollution may vary among population subgroups according to SES, educational level, air conditioning use, proximity to roadways, geographic location, level of physical activity, and work environment (Kraft et al. 2005). We analyzed several factors that may confer susceptibility and/or vulnerability to air pollution, and most of our results are in agreement with previous studies, even though some of the associations could be partially spurious because of the multiple tests performed on the same database.

Many chronic health conditions appeared to increase susceptibility to effects of NO_2_; in particular, people with previous hospital discharges for cardiovascular conditions appeared to be at higher risk. The estimated effect of NO_2_ was highest for subjects with three or more chronic health conditions. The estimated NO_2_ effect was stronger among the elderly—a finding that is consistent with previous studies for exposure to particles (Aga et al. 2003), although the association for those ≥ 85 years of age was not significantly different from that for the 35- to 64-year age group.

The results obtained in the present study do not clarify the role of SES as an effect modifier of the association between mortality and variation in NO_2_. However, data on SES were not available at an individual level (only at area level), and this represents a limitation of our study. Another limitation is related to the ascertainment of the chronic health condition, which we based on hospital discharge records and suffers from the limits of accuracy of the source used. Therefore, we tried to increase the sensitivity of the definition of chronic conditions by using all hospital admissions in the 2-year period before death and by considering both primary and contributory causes.

## Conclusion

This study confirms a clear association between short-term exposure to NO_2_ and natural mortality and supports increased susceptibility among people suffering from chronic cardiovascular conditions and diabetes. These conditions should be considered when developing prevention-oriented health policies (Künzli 2002).
